# Household transmission of SARS-CoV-2 from adult index cases living with and without HIV in South Africa, 2020-2021: a case-ascertained, prospective observational household transmission study

**DOI:** 10.1093/cid/ciac640

**Published:** 2022-08-04

**Authors:** Jackie Kleynhans, Sibongile Walaza, Neil A Martinson, Mzimasi Neti, Anne von Gottberg, Jinal N Bhiman, Dylan Toi, Daniel G Amoako, Amelia Buys, Kedibone Ndlangisa, Nicole Wolter, Leisha Genade, Lucia Maloma, Juanita Chewparsad, Limakatso Lebina, Linda de Gouveia, Retshidisitswe Kotane, Stefano Tempia, Cheryl Cohen

**Affiliations:** Centre for Respiratory Diseases and Meningitis, National Institute for Communicable Diseases of the National Health Laboratory Service, Johannesburg, South Africa. School of Public Health, Faculty of Health Sciences, University of the Witwatersrand, Johannesburg, South Africa; Centre for Respiratory Diseases and Meningitis, National Institute for Communicable Diseases of the National Health Laboratory Service, Johannesburg, South Africa. School of Public Health, Faculty of Health Sciences, University of the Witwatersrand, Johannesburg, South Africa; Perinatal HIV Research Unit (PHRU), University of the Witwatersrand, Johannesburg, South Africa. Johns Hopkins University Center for TB Research, Baltimore, Maryland, United States of America; Centre for Respiratory Diseases and Meningitis, National Institute for Communicable Diseases of the National Health Laboratory Service, Johannesburg, South Africa; Centre for Respiratory Diseases and Meningitis, National Institute for Communicable Diseases of the National Health Laboratory Service, Johannesburg, South Africa. School of Pathology, Faculty of Health Sciences, University of the Witwatersrand, Johannesburg, South Africa; Centre for Respiratory Diseases and Meningitis, National Institute for Communicable Diseases of the National Health Laboratory Service, Johannesburg, South Africa. School of Pathology, Faculty of Health Sciences, University of the Witwatersrand, Johannesburg, South Africa; Centre for Respiratory Diseases and Meningitis, National Institute for Communicable Diseases of the National Health Laboratory Service, Johannesburg, South Africa; Centre for Respiratory Diseases and Meningitis, National Institute for Communicable Diseases of the National Health Laboratory Service, Johannesburg, South Africa. School of Health Sciences, College of Health Sciences, University of KwaZulu-Natal, KwaZulu-Natal, South Africa; Centre for Respiratory Diseases and Meningitis, National Institute for Communicable Diseases of the National Health Laboratory Service, Johannesburg, South Africa; Centre for Respiratory Diseases and Meningitis, National Institute for Communicable Diseases of the National Health Laboratory Service, Johannesburg, South Africa; Centre for Respiratory Diseases and Meningitis, National Institute for Communicable Diseases of the National Health Laboratory Service, Johannesburg, South Africa. School of Pathology, Faculty of Health Sciences, University of the Witwatersrand, Johannesburg, South Africa; Perinatal HIV Research Unit (PHRU), University of the Witwatersrand, Johannesburg, South Africa; Perinatal HIV Research Unit (PHRU), University of the Witwatersrand, Johannesburg, South Africa; Perinatal HIV Research Unit (PHRU), University of the Witwatersrand, Johannesburg, South Africa; Perinatal HIV Research Unit (PHRU), University of the Witwatersrand, Johannesburg, South Africa. Africa Health Research Institute (AHRI), Durban, South Africa; Centre for Respiratory Diseases and Meningitis, National Institute for Communicable Diseases of the National Health Laboratory Service, Johannesburg, South Africa; Centre for Respiratory Diseases and Meningitis, National Institute for Communicable Diseases of the National Health Laboratory Service, Johannesburg, South Africa; Centre for Respiratory Diseases and Meningitis, National Institute for Communicable Diseases of the National Health Laboratory Service, Johannesburg, South Africa. School of Public Health, Faculty of Health Sciences, University of the Witwatersrand, Johannesburg, South Africa; Centre for Respiratory Diseases and Meningitis, National Institute for Communicable Diseases of the National Health Laboratory Service, Johannesburg, South Africa. School of Public Health, Faculty of Health Sciences, University of the Witwatersrand, Johannesburg, South Africa

**Keywords:** SARS-CoV-2, COVID-19, HIV, transmission, acquisition

## Abstract

**Background:**

In South Africa, 19% of the adult population are living with HIV (LWH). Few data on the influence of HIV on SARS-CoV-2 household transmission are available.

**Methods:**

We performed a case-ascertained, prospective household transmission study of symptomatic index SARS-CoV-2 cases LWH and HIV-uninfected adults and their contacts in South Africa, October 2020 to September 2021. Households were followed up thrice weekly for 6 weeks to collect nasal swabs for SARS-CoV-2 testing. We estimated household cumulative infection risk (HCIR) and duration of SARS-CoV-2 positivity (at cycle threshold value <30 as proxy for high viral load).

**Results:**

We recruited 131 index cases and 457 household contacts. HCIR was 59% (220/373); not differing by index HIV status (60% [51/85] in cases LWH vs 58% [163/279] in HIV-uninfected cases, OR 1.0, 95%CI 0.4-2.3). HCIR increased with index case age (35-59 years: aOR 3.4 95%CI 1.5-7.8 and ≥60 years: aOR 3.1, 95%CI 1.0-10.1) compared to 18-34 years, and contacts’ age, 13-17 years (aOR 7.1, 95%CI 1.5-33.9) and 18-34 years (aOR 4.4, 95%CI 1.0-18.4) compared to <5 years. Mean positivity duration at high viral load was 7 days (range 2-17), with longer positivity in cases LWH (aHR 0.4, 95%CI 0.1-0.9).

**Conclusions:**

Index HIV status was not associated with higher HCIR, but cases LWH had longer positivity duration at high viral load. Adults aged >35 years were more likely to transmit, individuals aged 13-34 to acquire SARS-CoV-2 in the household. As HIV infection may increase transmission, health services must maintain HIV testing and antiretroviral therapy initiation.

### Introduction

By January 2022, South Africa reported 3.6 million coronavirus disease 2019 (COVID-19) cases and 94.3 thousand deaths; the highest reported from Africa.[1, 2] South Africa experienced four severe acute respiratory syndrome coronavirus 2 (SARS-CoV-2) waves: the first dominated by ancestral variants, followed by Beta (B.1.351), Delta (B.1.617.2) and Omicron (B.1.1.529) variant waves, respectively.[3]

Although incident HIV infections and AIDS-related deaths from 2010 to 2019 in South Africa reduced by 53% and 61%, respectively, the burden of HIV is still high, with an estimated 19% of the adult population aged 15-49 living with HIV (LWH); the fourth highest in Sub-Saharan Africa.[4] The SARS-CoV-2 pandemic impacted several health programs, including HIV testing and care. During initial lockdowns there was a decline in HIV testing and antiretroviral therapy (ART) initiations, which gradually returned to pre-lockdown levels after the lockdown in South Africa[5] and other Sub-Saharan African countries.[6]

Few studies report the influence of HIV infection on SARS-CoV-2, with most data available from high income countries and little evidence from sub-Saharan Africa where most people LWH reside.[4] People LWH are at greater risk for hospitalisation[7-10] and death[9-13] when infected with SARS-CoV-2, but SARS-CoV-2 prevalence is similar between people LWH and HIV-uninfected individuals.[14] Risk for hospitalisation and death increases with decline in CD4+ T cells.[8, 9, 13] Limited data are available on the role of HIV in transmission of SARS-CoV-2. One study showed no increase in household transmission from, or acquisition of SARS-CoV-2 infection in people LWH.[2] People LWH with severe COVID-19 who are not virally suppressed shed SARS-CoV-2 for longer periods,[2, 15] which could lead to increased secondary transmission.

We assessed household cumulative infection risk (HCIR), duration of SARS-CoV-2 positivity (episode duration), serial interval in households with SARS-CoV-2 index cases LWH and HIV-uninfected during October 2020 to September 2021 during Beta and Delta waves.

### Methods

We conducted a case-ascertained, prospective observational household transmission study of household contacts of symptomatic adult index SARS-CoV-2 cases LWH and HIV-uninfected at two sites in South Africa, Klerksdorp (North West Province) and Soweto (Gauteng Province). Planned sample size was 264 and 176 contacts from households with an HIV-uninfected and HIV-infected index case, respectively (detailed in supplement). Actual sample size was 344 and 103 household members exposed to an HIV-uninfected and HIV-infected index case, respectively.

*Screening for index cases*

Screening procedures are detailed in supplementary methods. In short, nasopharyngeal swabs were collected from clinic attendees aged ≥18 years with symptom onset ≤5 days prior to screening and tested for SARS-CoV-2 using real-time reverse transcription polymerase chain reaction (rRT-PCR).

*Household enrolment*

We approached households of individuals who tested positive for SARS-CoV-2, with symptom onset less than 7 days prior, no household members reporting symptoms in 14 days prior to index screening. Households with ≥3 eligible members (sharing ≥2 meals in same residence for ≥2 days/week), of whom ≥70% consented to participate were enrolled. Households that withdrew within 10 days from index symptom start date were excluded from the analysis.

*Index and household follow-up*

We visited households thrice weekly for 6 weeks to collect nasal swabs, symptoms and healthcare seeking behaviour from consenting household members. At the first and last study visits, clotted blood was collected for serological testing. Follow-up started on 12 October 2020 and continued to 11 August 2021 and 28 September 2021 in Klerksdorp and Soweto, respectively.

*SARS-CoV-2 detection*

Nasopharyngeal (screening) and nasal (follow-up) specimens were tested for SARS-CoV-2 genes by qualitative rRT-PCR, using the Allplex™ 2019-nCoV kit (Seegene Inc., Seoul, South Korea). Specimens were considered positive for SARS-CoV-2 if the cycle threshold ($C_t$) value was <40 for any gene target.

*SARS-CoV-2 variants*

We characterized the first SARS-CoV-2 positive specimen for each participant on the Allplex™ SARS-CoV-2 Variants I and II PCR assays (Seegene Inc., Seoul, Korea) and through full genome sequencing on the Ion Torrent Genexus platform (Thermo Fisher Scientific, USA). We classified the infection episodes as Alpha, Beta, Delta, non-Alpha/Beta/Delta or unknown variant (detailed in supplement).

*Serology*

We used an in-house ELISA to detect antibodies against SARS-CoV-2 spike protein[18] and nucleocapsid protein using Roche Elecsys anti-SARS-CoV-2 assay. Individuals were considered seropositive if they tested positive on either.

*Statistical analysis*

Definitions used are in Table 1. To assess factors associated with HCIR we used logistic regression accounting for within site and household clustering using a mixed effects hierarchical regression model. To assess factors associated with a time to event analysis (serial interval and episode duration), we used a multilevel mixed-effects survival model with Weibull accelerated failure time analysis. Hazard ratios <1 correspond to longer episode duration than reference group used. Since multiple members from the same household could potentially be included in the serial interval analysis, we controlled for both site and household level clustering in in the analysis. In the episode duration analysis, we controlled for only site clustering (one index per household). In addition to using site to control for clustering, it was also included as a co-variate in models. Episode duration was assessed at any $C_t$ value (<40) and $C_t$<30 (proxy for high viral load based on virus culture studies [19]). We first assessed co-variates on univariate analysis, including all with p<0.2 in the multivariable analysis. We performed backwards elimination and kept all variables with $p<0.05$ in the final model, except those included *a priori*. We included site, SARS-CoV-2 variant and index immune suppression related to HIV status (defined as CD4+ T cell count <200 cells/ml) in the HCIR and episode duration models *a priori* irrespective of statistical significance in the multivariable model Site was included *a priori* in the serial interval analysis. Due to low SARS-CoV-2 vaccination coverage in study participants (only one contact, Table 2), vaccination status was not included in our analyses.

*Sensitivity analysis*

To assess the influence of loss to follow-up, we performed a sensitivity analysis including only households where 65% of enrolled household members completed 65% of follow-up visits in the first three weeks of follow-up. To explore the effect of previous SARS-CoV-2 infection, we considered all household members irrespective of baseline serology as susceptible contacts in the HCIR analysis.

*Ethics*

The study protocol was approved by University of the Witwatersrand Human Research Ethics Committee (Reference M2008114). Participants in follow-up received grocery store vouchers of USD 3 per visit to compensate for time required for specimen collection and interview.

### Results

*Screening, enrolment and follow-up*

From 2 October 2020 to 30 September 2021, we screened 1,531 clinic attendees for SARS-CoV-2;18% (277) tested positive on rRT-PCR. Of those testing positive who met eligibility criteria for household enrolment (n=277), 143 (52%) households were approached and 131 (92%) were enrolled. Reasons for non-inclusion shown in Figure 1. The final cohort consisted of 131 index cases and 457 household contacts (Figure 1), median household size of 4.

Twenty-one percent (28/131) of index cases were LWH, and two index cases initially agreed but then refused HIV testing after enrolment (classified as HIV unknown during analyses). The majority (93/131, 71%) of index cases and contacts (265/457, 58%) were female (Table 2).

Of 10,584 potential study visits to individual participants, we completed 8,509 (80%) visits, and detected SARS-CoV-2 in 17% (1,454/8,352) of nasal swabs collected (Supplementary Figure 1).

*Secondary SARS-CoV-2 cases*

We diagnosed 232 (51%) rRT-PCR-confirmed secondary cases from 457 contacts linked to 131 index cases. One third (69/232) of secondary cases reported ≥1 symptom during their SARS-CoV-2 episode, reporting on average 3 symptoms (range 1-9). The mean symptom duration was 11 days (range 4-40). The most common symptoms reported were cough (45/69, 65%), headache (31/67, 46%) and fever (27/69, 39%). Five secondary cases were hospitalized (Supplementary Table 5).

*Household cumulative infection risk*

Of 131 households, we excluded seven (5%) households from HCIR analysis: four (13 contacts) had SARS-CoV-2 clusters with >1 variant detected (Supplementary Figure 2), and in three (8 contacts) all contacts were seropositive at baseline with no rRT-PCR-confirmed SARS-CoV-2 infection during follow-up. An additional 42 contacts were excluded because they had prevalent SARS-CoV-2 antibodies at baseline, and no SARS-CoV-2 detection during follow-up. We therefore included 124/131 (95%) index cases with 373/436 (86%) contacts for this analysis.

The HCIR was 59% (220/373) overall. Mean number of household contacts testing positive for SARS-CoV-2 following index episode was 2 (range 0 to 7). On univariate analysis, HCIR was similar in households with HIV-uninfected index cases (58%, 173/293) and households where index was LWH (60%, 50/83, OR 1.0, 95%CI 0.4-2.3).

On multivariable analysis, after adjusting for site and immune suppression, factors associated with household transmission were index case aged 35-59 years (aOR 3.4 95% CI 1.5-7.8) and ≥60 years (aOR 3.1, 95%CI 1.0-10.1) compared to 18-34 years; index cases with a $C_t$ value <25 (aOR 5.3, 95%CI 1.6-17.6) and 25-35 (aOR 7.5, 95%CI 2.2-26.0) compared to $C_t$>35; infection with Delta variant compared to Beta (aOR 4.6, 95%CI 1.5-14.4, Figure 4, Supplementary table 1).

Fourteen percent (17/124) of index cases were LWH, not immune suppressed, while 3% (4/124) were LWH, immune suppressed. Contacts of index cases LWH with immune suppression, had higher HCIR (62%, 8/13) compared to contacts of index cases not immune suppressed (58%, 30/52), but was not statistically significant on multivariable analysis (aOR 2.5, 95%CI 0.4-15.3). Contact age 13-17 years (aOR 7.1, 95%CI 1.5-33.9) and 18-34 years (aOR 4.4, 95%CI 1.0-18.4) compared to <5 years and contacts not currently smoking (aOR 3.9, 95%CI 1.7-9.0) were associated with higher HCIR.

*Episode duration*

We right-censored 6% of (8/131) index cases who were SARS-CoV-2 positive on their last specimen collected at the end of follow-up (n=5) or at withdrawal (n=3). When including all 131 index cases, mean episode duration for index cases was 20 days (range 3-47, Figure 2). When excluding the eight right-censored individuals (n=123), mean episode duration for index cases was 19 days (range 3 to 45). Mean episode duration was similar for HIV-uninfected index cases (20 days, range 3-45) compared to index cases LWH (17 days, range 3-45, HR 0.8, 95%CI 0.5-1.2).

On multivariable analysis, factors associated with longer episode duration in days, was Soweto site (aHR 0.5, 95%CI 0.3-0.7), being 35-59 years old (aHR 0.4, 95%CI 0.2-0.6) and ≥60 years (aHR 0.2, 95%CI 0.1-0.5) compared to 18-34 years, $C_t$<25 (aHR 0.6, 95%CI 0.2-0.9) compared to $C_t$>35 (Figure 5, Supplementary table 2) and being seropositive at end of follow-up (aHR 0.1, 95%CI 0.0-0.3). Individuals infected with Delta variant (aHR 2.6, 95%CI 1.4-5.0) and a non-variant of concern (aHR 4.0, 95%CI 1.9-8.2), compared to Beta, had shorter episode durations (Figure 5, Supplementary Table 2).

Eighty-eight (67%) of index cases had ≥1 specimen with ≥1 target with $C_t$<30. Mean episode duration with $C_t$<30 was 7 days (range 2-17). On multivariable analysis, factors associated with longer episode duration considering only specimens with at least one target with a $C_t$<30, was female sex (aHR 0.5, 95%CI 0.3-0.9), LWH (aHR 0.4, 95%CI 0.1-0.9) and being seropositive at the end of follow-up (aHR 0.01, 95%CI 0.001-0.2, (Figure 5, Supplementary Table 3).

*Serial interval*

We excluded 5 index contact pairs where serial interval was >21 days; three with an HIV-uninfected index and two pairs where the index was LWH. Mean serial interval for index cases and symptomatic contact pairs included in the risk factor analysis was 6 days (range 1-20, Figure 3). Mean serial interval for HIV-uninfected index cases was 6 days (range 1-15) and for index cases LWH 8 days (range 2-20).

On multivariable analysis, pairs with contacts aged 35-59 years (aHR 0.3, 95%CI 0.1-0.9) and ≥60 years (aHR 0.2, 95%CI 0.0-0.8), compared to being aged 18-34 years, and where the contact was LWH (aHR 0.1, 95%CI 0.0-0.8) had longer serial intervals (Figure 6, Supplementary Table 4).

*Sensitivity analysis*

When not excluding individuals seropositive at baseline with no rRT-PCR-confirmed SARS-CoV-2 infection during follow-up, HCIR was 51% (220/436) overall. We found similar factors associated with HCIR (Supplementary Table 2). When only including households where 65% of members completed 65% of visits, we included 112 index cases with 342 contacts. Factors associated with HCIR, episode duration and serial interval were similar to what we observed in the main analysis (Supplementary Tables 6-9)

### Discussion

We performed a case-ascertained, prospective household transmission study for SARS-CoV-2 in South Africa, including 131 index cases, 28 of whom were LWH; and 457 household contacts. We observed a

59% HCIR, HCIR being higher in households with older index cases and contacts aged 13-17 years and 18-34 years. HCIR was also higher in households with Delta-infected index cases vs Beta. The HCIR was similar in index cases LWH and HIV-uninfected index cases. Index episode durations were longer in older aged individuals. Episode duration at high viral load ($C_t$<30) was longer in index cases LWH and serial interval was longer in contacts LWH.

HCIR from previous studies have varied based on study design, symptom status of index, timing within the epidemic[20] and SARS-CoV-2 variant.[16] In our study only including symptomatic index cases, we estimated the HCIR at 59%, a higher estimate than the overall 37% reported from a recent meta-analysis which included 33 studies performed in 2021 and 2022, and the variant-specific 23% and 30% estimates for Beta and Delta variants, respectively.[16] In a Madagascar study including both symptomatic and non-symptomatic index cases, HCIR was 39%.[21] The higher estimate seen in our study may be influenced by symptom severity, as proposed in previous studies,[22] or the inclusion of only adult index cases; adult index cases result in higher HCIR.[23] In our study, households with index cases older than 35 years were three times more likely to result in higher HCIR compared to when index cases were aged 18-34 years. HCIR was also higher when contacts were aged 13-17 and 18-34, compared to <5 years. Contacts aged 13-18 years were also associated with higher HCIR in the South African PHIRST-C study,[2] although studies from earlier in the pandemic showed higher attack rates in elderly household members.[22, 24] This may be related to the shift in age-distribution of cases from the older population to younger individuals with progression of the pandemic.[2, 25, 26] As seen previously,[2, 27, 28] we also observed higher secondary attack rates where the minimum rRT-PCR $C_t$ was lower for the index case, which could be considered a proxy for higher viral load.

We observed no difference in HCIR in households with index cases living with and without HIV. However, we observed a higher HCIR in people living with HIV (PLWH) who were immune suppressed, but this association was not statistically significant possibly due to low numbers (n=14) of included immunosuppressed index cases LWH. This would fit with previous studies finding that immunocompromised PLWH that shed virus at low $C_t$ value for longer[2, 15] allowing longer opportunity for secondary infections, although we did not observe increases transmission in our study, possibly due to small numbers.

The mean episode duration in index cases of 19 days, was higher than the 11 days reported from the household cohort study from South Africa[2] but more similar to the 18 days estimate from a meta-analysis for viral shedding time.[29] This may be due to our analysis being limited to symptomatic individuals, shown to be associated with longer episode duration.[2, 29] Episode duration in this study is also higher than studies on hospitalised South African patients where median episode duration was 13 days[15]. Previous studies from South Africa in the community and in hospitals found that immunocompromised people LWH shed SARS-CoV-2 for longer.[2, 15] While we did not find overall longer shedding in people LWH, when considering detection at $C_t$<30 (proxy for high viral load), we also observed people LWH had longer episode durations. Longer episode durations may allow increased opportunity for viral evolution, and the establishment of novel variants [30].

Previous serial interval estimates ranged between 4 to 7.5 days[2, 22],similar to our estimate of 6 days. We did not find any index characteristics related to longer serial intervals, but longer serial intervals were observed in contacts older than 35 years, and those LWH. Individuals with compromised immune systems (PLWH, elderly) may still be able to be infected with SARS-CoV-2 towards the end of the index episode when viral loads are lower. Due to their increased risk for hospitalization and death [9, 13], there should be continued support for prioritising COVID-19 vaccination in these populations.

Our study had limitations. We assumed the first household member presenting with symptoms was the index case. If the true index cases were asymptomatic, we would have underestimated the serial interval, although HCIR estimates should not be greatly affected. Due to the delay between index screening and household enrolment, we did not have the exact date of first SARS-CoV-2 positivity in household contacts, and may have also over-estimated HCIR if there were multiple introductions of SARS-CoV-2 in the household. By excluding individuals seropositive at baseline with no SARS-CoV-2 infection during follow-up, we assumed 100% protection from previous infections, which is likely not correct, and may have overestimated HCIR. When including these individuals, HCIR reduced by 8%. We were unable to reach the planned sample size for contacts of index cases LWH, and may have been underpowered to detect some differences. By only including symptomatic index cases, our results may not be generalizable to households with asymptomatic infections. We did not consider the role of age-related contact patterns within the household on household transmission. This should be considered for future studies.

In conclusion, in two communities in South Africa, HCIR was higher than in previous studies[16] and not influenced by HIV status. Episode duration at high viral loads (inferred by $C_t$<30) was increased for index cases LWH, which may lead to increased risk for secondary transmission and viral evolution. Serial interval was longer in contacts LWH. Although these findings indicate that HIV status of the index case did not affect SARS-CoV-2 transmission to household contacts, it may still play a role, especially if PLWH are not virally suppressed. Sustaining and strengthening HIV treatment and care programs should be a focus moving forward to ensure PLWH are diagnosed and virally suppressed to reduce prolonged shedding of SARS-CoV-2 and potentially reduce increased transmission, as well as their risk for hospitalisation and death [9].

### NOTES

#### Author contributions

Conception and design of study: JK, SW, NAM, AvG, JNB, MW, LL, ST, CC

Data collection and laboratory processing: AvG, JNB, DGA, AB, KN, NW, LG, LN, JC, LdG, RK

Analysis and interpretation: JK, SW, ST, CC

Accessed and verified underlying data: JK, MN, LG, LM, JC

Drafted the Article: JK

All authors critically reviewed the Article. All authors had access to all the data reported in the study.

### Acknowledgements

All individuals participating in the study, field teams for their hard work and dedication to the study, the CRDM laboratory team, Andrew Whitelaw, June Fabian, Themba Radebe and Thoko Sifunda from the safety committee.

This study protocol was adapted from the World Health Organization Unity Studies Early Investigation Protocol for Household transmission investigation protocol for COVID-19 infection.

### Data availability

The investigators welcome enquiries about possible collaborations and requests for access to the dataset. Data will be shared after approval of a proposal and with a signed data access agreement. Investigators interested in more details about this study, or in accessing these resources, should contact the corresponding author.

### Funding

This research was funded by the Wellcome Trust (Grant number 221003/Z/20/Z) in collaboration with the Foreign, Commonwealth and Development Office, United Kingdom. For the purpose of open access, the author has applied a CC BY-ND public copyright licence to any Author Accepted Manuscript version arising from this submission. AvG, JK, and SW also report grants paid to institution in support of this work from WHO AFRO, Africa Pathogen Genomics Initiative (Africa PGI) through African Society of Laboratory Medicine (ASLM) and Africa CDC, US Centers for Disease Control and Prevention, South African Medical Research Council (SAMRC), and UK Department of Health and Social Care and managed by the Fleming Fund: SEQAFRICA project. LL and NW also report support for this work from the US Centre for Disease Control and Prevention. MN reports support for this work paid to institution from America Centre for Disease Control. NAM reports support for this work paid to institution from the National Institute of Communicable Diseases.

### Potential conflicts of interest

CC has received grant support from Sanofi Pasteur, US Centers for Disease Control and Prevention, Welcome Trust, Programme for Applied Technologies in Health (PATH), Bill & Melinda Gates Foundation and South African Medical Research Council (SA-MRC). NW, AvG , JK,and SW report receiving grant funds paid to institution from Sanofi Pasteur and Bill & Melinda Gates Foundation. AvG also reports an unpaid position as Chairperson of National Advisory Group on Immunisation. LL reports receiving grant funds from US Centers for Disease Control and Prevention, and Bill & Melinda Gates Foundation. NM reports receiving grant funds paid to institution from Pfizer to conduct observational studies into the burden of pneumonia in South Africa; unpaid participation on a data safety monitoring board of a TB HDT trial and Scientific Advisory Board on a trial of a electronic reminder to take daily TB treatment; and unpaid leadership or fiduciary role with Setshaba Research Center. SW also reports an unpaid leadership or fiduciary role in other board, society, committee or advocacy group. All other authors have no competing/conflict of interest.

## Tables

**Table 1.** Definitions

| | |
|---|---|
| Household | A group of three or more people who regularly share at least two meals in the same residence at least two days per week (residential institutions excluded). |
| Index case | The first household member who had COVID-like-symptoms. We assumed that the household member screened was the index case within the household as they were the first household member to develop symptoms |
| SARS-CoV-2 infection episode | At least one nasal swab rRT-PCR positive for SARS-CoV-2. Individuals who seroconverted during follow-up but with no rRT-PCR-confirmed infection were not included in secondary case analyses. |
| SARS-CoV-2 cluster | Composed of all infections within a household within an interval between infections of ≤2 weeks including single infections within a household. |
| Episode duration | Duration of SARS-CoV-2 positivity. The start of symptom onset to the midpoint between the last positive swab and first negative. Individuals who were still SARS-CoV-2 positive on the last study visit (whether at end of follow-up or due to early withdrawal) were right-censored for the multivariable analysis. |
| Serial interval | Number of days between the onset of symptoms in the index case, to the onset of symptoms in the secondary case. The serial interval was the. Multivariable analyses were restricted to symptomatic secondary cases, and restricted to serial interval periods of ≤21 days as longer serial intervals could have been due to tertiary cases or secondary infections. |
| Household cumulative infection risk (HCIR) | The percentage of susceptible household members (based on baseline serology) that had at least one SARS-CoV-2 positive swab from the start of follow-up, up to two weeks from the last SARS-CoV-2 positive swab of the index case. Considering susceptibility was important because following the second wave of SARS-CoV-2 infection in South Africa, 41% of individuals were estimated to have had previous SARS-CoV-2 infection.[26] Individuals with SARS-CoV-2 antibodies detected at baseline, but also tested positive on rRT-PCR, were included in the HCIR calculation. Individuals for whom no baseline serology was available were included in the analysis as presumed susceptible. We did not consider any secondary introductions in the household for our analysis. Households where members had SARS-CoV-2 infection with different variants of concern were excluded from the analysis. |

**Table 2.** Baseline characteristics of SARS-CoV-2 index cases (n=131) and their household contacts (n=457), Klerksdorp and Soweto, South Africa, September 2020 – October 2021.

| Characteristic | Overall | | Index Case, n/N (%) | | Household contact, n/N (%) | |
|---|---|---|---|---|---|---|
| | Index Case, n/N (%) | Household contact, n/N (%) | Klerksdorp | Soweto | Klerksdorp | Soweto |
| **Household Characteristics** | | | | | | |
| **Index case/contact** | 131/588 (22) | 457/588 (78) | 62/274 (23) | 69/314 (22) | 212/274 (77) | 245/314 (78) |
| **Household size** | | | | | | |
| 3-5 | 106/131 (81) | 305/457 (67) | 51/62 (82) | 55/69 (80) | 146/212 (69) | 159/245 (65) |
| 6-10 | 25/131 (19) | 152/457 (33) | 11/62 (18) | 14/69 (20) | 66/212 (31) | 86/245 (35) |
| **Rooms used for sleeping** | | | | | | |
| 1-2 | 66/117 (56) | 209/401 (52) | 33/57 (58) | 33/60 (55) | 114/192 (59) | 95/209 (45) |
| 3-4 | 42/117 (36) | 141/401 (35) | 22/57 (39) | 20/60 (33) | 69/192 (36) | 72/209 (34) |
| >4 | 9/117 (8) | 51/401 (13) | 2/57 (4) | 7/60 (12) | 9/192 (5) | 42/209 (20) |
| **Crowding** | 89/131 (68) | 330/457 (72) | 43/62 (69) | 46/69 (67) | 158/212 (75) | 172/245 (70) |
| **Child <5 years** | 17/131 (13) | 63/457 (14) | 10/62 (16) | 7/69 (10) | 43/212 (20) | 20/245 (8) |
| **HH member smokes inside** | 30/131 (23) | 93/457 (20) | 18/62 (29) | 12/69 (17) | 51/212 (24) | 42/245 (17) |
| **Main water source inside home** | 92/131 (70) | 321/457 (70) | 38/62 (61) | 54/69 (78) | 124/212 (58) | 197/245 (80) |
| **Place to wash hands inside** | 129/131 (98) | 451/457 (99) | 62/62 (100) | 67/69 (97) | 212/212 (100) | 239/245 (98) |

| Characteristic | Overall | | Index Case, n/N (%) | | Household contact, n/N (%) | |
|---|---|---|---|---|---|---|
| | Index Case, n/N (%) | Household contact, n/N (%) | Klerksdorp | Soweto | Klerksdorp | Soweto |
| **Main cooking fuel** | | | | | | |
| Electricity | 127/131 (97) | 444/457 (97) | 58/62 (94) | 69/69 (100) | 199/212 (94) | 245/245 (100) |
| Gas/Paraffin | 4/131 (3) | 13/457 (3) | 4/62 (6) | 0/69 (0) | 13/212 (6) | 0/245 (0) |
| **Monthly household income** | | | | | | |
| <R400 | 5/131 (4) | 17/457 (4) | 4/62 (6) | 1/69 (1) | 15/212 (7) | 2/245 (1) |
| R401 to R800 | 6/131 (5) | 19/457 (4) | 4/62 (6) | 2/69 (3) | 12/212 (6) | 7/245 (3) |
| R801 to R1 600 | 12/131 (9) | 39/457 (9) | 10/62 (16) | 2/69 (3) | 33/212 (16) | 6/245 (2) |
| R1 601 to R3 200 | 23/131 (18) | 86/457 (19) | 12/62 (19) | 11/69 (16) | 43/212 (20) | 43/245 (18) |
| R3 201 to R6 400 | 21/131 (16) | 68/457 (15) | 7/62 (11) | 14/69 (20) | 23/212 (11) | 45/245 (18) |
| R6 401 to R12 800 | 8/131 (6) | 33/457 (7) | 2/62 (3) | 6/69 (9) | 5/212 (2) | 28/245 (11) |
| R12 801 to R25 600 | 6/131 (5) | 19/457 (4) | 0/62 (0) | 6/69 (9) | 0/212 (0) | 19/245 (8) |
| Refused to disclose | 50/131 (38) | 176/457 (39) | 23/62 (37) | 27/69 (39) | 81/212 (38) | 95/245 (39) |
| **Individual characteristics** | | | | | | |
| **Age** | | | | | | |
| <5 | | 19/457 (4) | | | 11/212 (5) | 8/245 (3) |
| 5-12 | | 80/457 (18) | | | 39/212 (18) | 41/245 (17) |

| Characteristic | Overall | | Index Case, n/N (%) | | Household contact, n/N (%) | |
|---|---|---|---|---|---|---|
| | Index Case, n/N (%) | Household contact, n/N (%) | Klerksdorp | Soweto | Klerksdorp | Soweto |
| 13-17 | | 70/457 (15) | | | 38/212 (18) | 32/245 (13) |
| 18-34 | 37/131 (28) | 126/457 (28) | 21/62 (34) | 16/69 (23) | 54/212 (25) | 72/245 (29) |
| 35-59 | 76/131 (58) | 113/457 (25) | 37/62 (60) | 39/69 (57) | 50/212 (24) | 63/245 (26) |
| ≥60 | 18/131 (14) | 49/457 (11) | 4/62 (6) | 14/69 (20) | 20/212 (9) | 29/245 (12) |
| **Sex** | | | | | | |
| Male | 38/131 (29) | 192/457 (42) | 17/62 (27) | 21/69 (30) | 93/212 (44) | 99/245 (40) |
| Female | 93/131 (71) | 265/457 (58) | 45/62 (73) | 48/69 (70) | 119/212 (56) | 146/245 (60) |
| **Level of education*** | | | | | | |
| No schooling | 6/129 (5) | 15/283 (5) | 3/60 (5) | 3/69 (4) | 1/120 (1) | 14/163 (9) |
| Primary | 4/129 (3) | 21/283 (7) | 2/60 (3) | 2/69 (3) | 14/120 (12) | 7/163 (4) |
| Secondary | 43/129 (33) | 89/283 (31) | 25/60 (42) | 18/69 (26) | 47/120 (39) | 42/163 (26) |
| Matriculation | 69/129 (53) | 136/283 (48) | 28/60 (47) | 41/69 (59) | 51/120 (43) | 85/163 (52) |
| Post-secondary | 7/129 (5) | 22/283 (8) | 2/60 (3) | 5/69 (7) | 7/120 (6) | 15/163 (9) |
| **Employment*** | | | | | | |
| Unemployed | 55/121 (45) | 159/258 (62) | 25/57 (44) | 30/64 (47) | 68/111 (61) | 91/147 (62) |
| Student | 10/121 (8) | 30/258 (12) | 5/57 (9) | 5/64 (8) | 11/111 (10) | 19/147 (13) |

| Characteristic | Overall: Index Case, n/N (%) | Overall: Household contact, n/N (%) | Index Case, n/N (%): Klerksdorp | Index Case, n/N (%): Soweto | Household contact, n/N (%): Klerksdorp | Household contact, n/N (%): Soweto |
|---|---|---|---|---|---|---|
| Employed | 56/121 (46) | 69/258 (27) | 27/57 (47) | 29/64 (45) | 32/111 (29) | 37/147 (25) |
| **Smoking cigarettes†** | 17/129 (13) | 66/323 (20) | 11/60 (18) | 6/69 (9) | 35/145 (24) | 31/178 (17) |
| **Smoke indoors** | 5/17 (29) | 15/66 (23) | 5/11 (45) | 0/6 (0) | 14/35 (40) | 1/31 (3) |
| **HIV status** | | | | | | |
| HIV-uninfected | 101/131 (77) | 176/457 (39) | 51/62 (82) | 50/69 (72) | 130/212 (61) | 46/245 (19) |
| HIV-infected | 28/131 (21) | 35/457 (8) | 10/62 (16) | 18/69 (26) | 16/212 (8) | 19/245 (8) |
| HIV status unknown | 2/131 (2) | 246/457 (54) | 1/62 (2) | 1/69 (1) | 66/212 (31) | 180/245 (73) |
| **HIV infected index in household** | | | | | | |
| Index HIV negative | 101/131 (77) | 344/457 (75) | 51/62 (82) | 50/69 (72) | 175/212 (83) | 169/245 (69) |
| Index HIV positive | 28/131 (21) | 103/457 (23) | 10/62 (16) | 18/69 (26) | 34/212 (16) | 69/245 (28) |
| Index HIV unknown | 2/131 (2) | 10/457 (2) | 1/62 (2) | 1/69 (1) | 3/212 (1) | 7/245 (3) |
| **CD4+ T cell count** | | | | | | |
| HIV-infected, not immune suppressed‡ | 17/28 (61) | 13/35 (37) | 5/10 (50) | 12/18 (67) | 2/16 (13) | 11/19 (58) |
| HIV-infected, immune suppressed‡ | 4/28 (14) | 2/35 (6) | 1/10 (10) | 3/18 (17) | 1/16 (6) | 1/19 (5) |
| HIV-infected no CD4+ T cell count | 7/28 (25) | 20/35 (57) | 4/10 (40) | 3/18 (17) | 13/16 (81) | 7/19 (37) |
| **Underlying illness§** | 32/129 (25) | 57/448 (13) | 12/60 (20) | 20/69 (29) | 205/212 (97) | 243/245 (99) |

| Characteristic | Overall | | Index Case, n/N (%) | | Household contact, n/N (%) | |
| --- | --- | --- | --- | --- | --- | --- |
| | Index Case, n/N (%) | Household contact, n/N (%) | Klerksdorp | Soweto | Klerksdorp | Soweto |
| **Body-mass index** | | | | | | |
| Underweight | 3/129 (2) | 29/448 (6) | 1/60 (2) | 2/69 (3) | 14/205 (7) | 15/243 (6) |
| Normal | 36/129 (28) | 206/448 (46) | 18/60 (30) | 18/69 (26) | 108/205 (53) | 98/243 (40) |
| Overweight | 25/129 (19) | 103/448 (23) | 10/60 (17) | 15/69 (22) | 39/205 (19) | 64/243 (26) |
| Obese | 65/129 (50) | 110/448 (25) | 31/60 (52) | 34/69 (49) | 44/205 (21) | 66/243 (27) |
| **Previous TB** | 7/129 (5) | 9/448 (2) | 2/60 (3) | 5/69 (7) | 6/205 (3) | 3/243 (1) |
| **Current TB** | 4/129 (3) | 2/448 (0) | 2/60 (3) | 2/69 (3) | 0/205 (0) | 2/243 (1) |
| **COVID-19 vaccination¶** | 0/131 (0) | 1/457 (0) | 0/62 (0) | 0/69 (0) | 1/212 (0) | 0/245 (0) |

*Individuals ≥18 years old. †Individuals ≥15 years old. ‡Immune suppressed defined as CD4+ T cell count <200 cells/ml §Underlying medical conditions: Self-reported history of diabetes, hypertension, asthma, lung disease, heart disease, stroke, spinal cord injury, epilepsy, cancer, liver disease, renal disease, pre-maturity. ¶At least one dose administered 14 days prior to enrolment.

### Figures

**Figure 1.** Adult SARS-CoV-2 index cases and household contacts enrolled, Klerksdorp and Soweto, South Africa, 2020-2021.

**Figure 2.** SARS-CoV-2 episode duration in HIV-infected and HIV-uninfected index cases, Klerksdorp and Soweto, South Africa, 2020-2021, (n=123). Excludes those positive at last specimen collected.

**Figure 3.** Interval between onset of symptoms in the index case and onset of symptoms in household contacts with SARS-CoV-2 (serial interval) by HIV-infection status of the index case, Klerksdorp and Soweto, South Africa, 2020-2021, (n=69). Arrow indicates cut-off for inclusion in analysis for factors associated with serial interval duration

**Figure 4.** Factors associated with SARS-CoV-2 household transmission in index cases and acquisition in household contacts on multivariable logistic regression, Klerksdorp and Soweto, South Africa, 2020-2021, (n=373).

**Figure 5.** Factors associated with SARS-CoV-2 episode duration (irrespective of $C_t$ value left, $C_t$<30 right) in index cases on Weibull accelerated failure time regression, Klerksdorp and Soweto, South Africa, 2020-2021, (n=123).

**Figure 6.** Factors associated with serial interval of SARS-CoV-2 in cases with symptomatic illness on Weibull accelerated failure time regression, Klerksdorp and Soweto, South Africa, 2020-2021, (n=62).

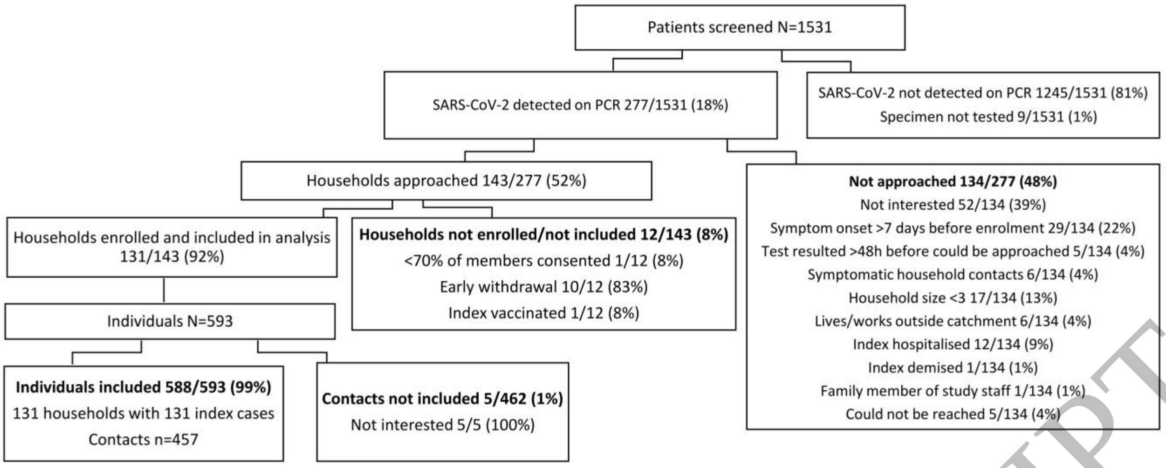

**Figure 1**
**292x112 mm (0.9 x  DPI)**

**Figure 2**
**130x80 mm (0.9 x  DPI)**

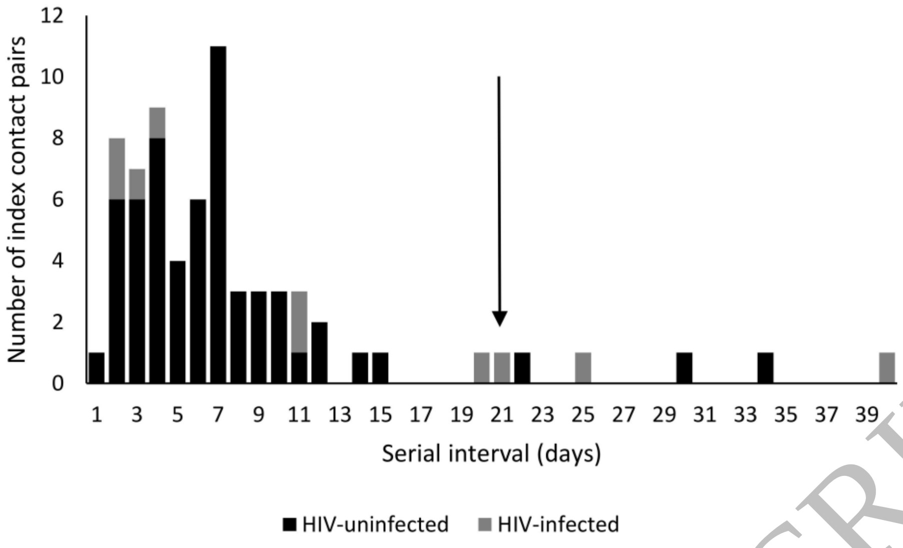

**Figure 3**
**130x80 mm (0.9 x DPI)**

**Figure 4**
**140x102 mm (0.9 x DPI)**

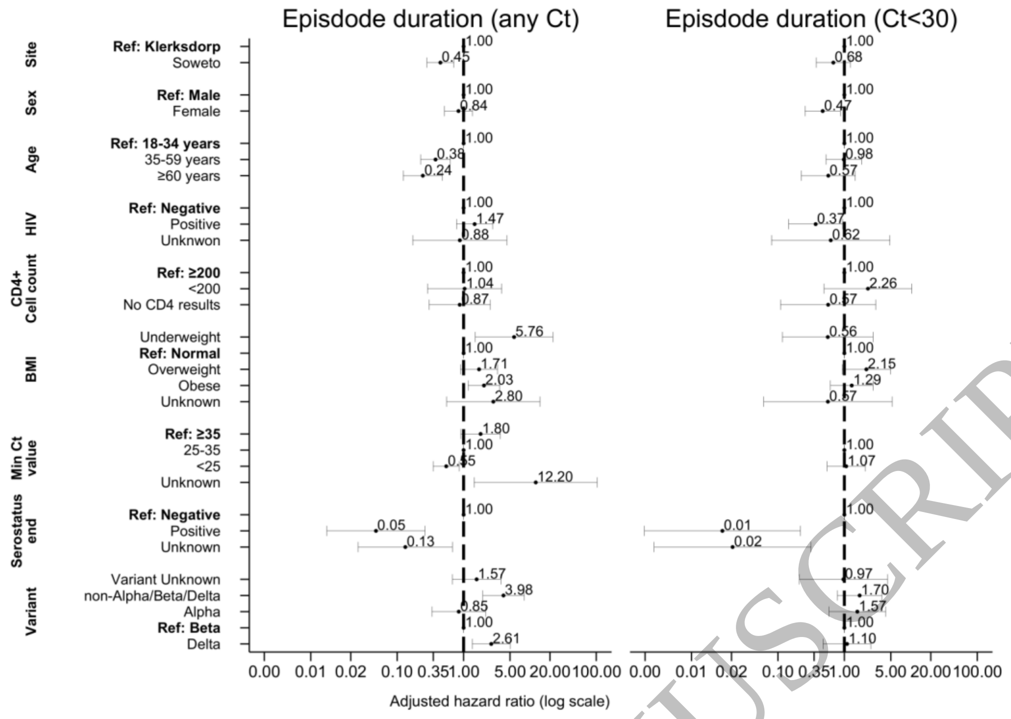

**Figure 5**
**140x102 mm (0.9 x DPI)**

**Figure 6**
**140x102 mm (0.9 x DPI)**

## Supplementary Material

ciac640_Supplementary_Data

