# Household Transmission of Severe Acute Respiratory Syndrome Coronavirus 2 From Adult Index Cases With and Without Human Immunodeficiency Virus in South Africa, 2020–2021: A Case-Ascertained, Prospective, Observational Household Transmission Study

**DOI:** 10.1093/cid/ciac640

**Published:** 2022-09-05

**Authors:** Jackie Kleynhans, Sibongile Walaza, Neil A Martinson, Mzimasi Neti, Anne von Gottberg, Jinal N Bhiman, Dylan Toi, Daniel G Amoako, Amelia Buys, Kedibone Ndlangisa, Nicole Wolter, Leisha Genade, Lucia Maloma, Juanita Chewparsad, Limakatso Lebina, Linda de Gouveia, Retshidisitswe Kotane, Stefano Tempia, Cheryl Cohen

**Affiliations:** Centre for Respiratory Diseases and Meningitis, National Institute for Communicable Diseases of the National Health Laboratory Service, Johannesburg, South Africa; School of Public Health, Faculty of Health Sciences, University of the Witwatersrand, Johannesburg, South Africa; Centre for Respiratory Diseases and Meningitis, National Institute for Communicable Diseases of the National Health Laboratory Service, Johannesburg, South Africa; School of Public Health, Faculty of Health Sciences, University of the Witwatersrand, Johannesburg, South Africa; Perinatal HIV Research Unit, University of the Witwatersrand, Johannesburg, South Africa; Center for TB Research, Johns Hopkins University, Baltimore, Maryland, USA; Centre for Respiratory Diseases and Meningitis, National Institute for Communicable Diseases of the National Health Laboratory Service, Johannesburg, South Africa; Centre for Respiratory Diseases and Meningitis, National Institute for Communicable Diseases of the National Health Laboratory Service, Johannesburg, South Africa; School of Pathology, Faculty of Health Sciences, University of the Witwatersrand, Johannesburg, South Africa; Centre for Respiratory Diseases and Meningitis, National Institute for Communicable Diseases of the National Health Laboratory Service, Johannesburg, South Africa; School of Pathology, Faculty of Health Sciences, University of the Witwatersrand, Johannesburg, South Africa; Centre for Respiratory Diseases and Meningitis, National Institute for Communicable Diseases of the National Health Laboratory Service, Johannesburg, South Africa; Centre for Respiratory Diseases and Meningitis, National Institute for Communicable Diseases of the National Health Laboratory Service, Johannesburg, South Africa; School of Health Sciences, College of Health Sciences, University of KwaZulu-Natal, KwaZulu-Natal, South Africa; Centre for Respiratory Diseases and Meningitis, National Institute for Communicable Diseases of the National Health Laboratory Service, Johannesburg, South Africa; Centre for Respiratory Diseases and Meningitis, National Institute for Communicable Diseases of the National Health Laboratory Service, Johannesburg, South Africa; Centre for Respiratory Diseases and Meningitis, National Institute for Communicable Diseases of the National Health Laboratory Service, Johannesburg, South Africa; School of Pathology, Faculty of Health Sciences, University of the Witwatersrand, Johannesburg, South Africa; Perinatal HIV Research Unit, University of the Witwatersrand, Johannesburg, South Africa; Perinatal HIV Research Unit, University of the Witwatersrand, Johannesburg, South Africa; Perinatal HIV Research Unit, University of the Witwatersrand, Johannesburg, South Africa; Perinatal HIV Research Unit, University of the Witwatersrand, Johannesburg, South Africa; Africa Health Research Institute, Durban, South Africa; Centre for Respiratory Diseases and Meningitis, National Institute for Communicable Diseases of the National Health Laboratory Service, Johannesburg, South Africa; Centre for Respiratory Diseases and Meningitis, National Institute for Communicable Diseases of the National Health Laboratory Service, Johannesburg, South Africa; Centre for Respiratory Diseases and Meningitis, National Institute for Communicable Diseases of the National Health Laboratory Service, Johannesburg, South Africa; School of Public Health, Faculty of Health Sciences, University of the Witwatersrand, Johannesburg, South Africa; Centre for Respiratory Diseases and Meningitis, National Institute for Communicable Diseases of the National Health Laboratory Service, Johannesburg, South Africa; School of Public Health, Faculty of Health Sciences, University of the Witwatersrand, Johannesburg, South Africa

**Keywords:** SARS-CoV-2, COVID-19, HIV, transmission, acquisition

## Abstract

**Background:**

In South Africa, 19% of adults are living with human immunodeficiency virus (HIV; LWH). Few data on the influence of HIV on severe acute respiratory syndrome coronavirus 2 (SARS-CoV-2) household transmission are available.

**Methods:**

We performed a case-ascertained, prospective household transmission study of symptomatic adult index SARS-CoV-2 cases LWH and not living with HIV (NLWH) and their contacts from October 2020 to September 2021. Households were followed up 3 times a week for 6 weeks to collect nasal swabs for SARS-CoV-2 testing. We estimated household cumulative infection risk (HCIR) and duration of SARS-CoV-2 positivity (at a cycle threshold value <30 as proxy for high viral load).

**Results:**

HCIR was 59% (220 of 373), not differing by index HIV status (60% LWH vs 58% NLWH). HCIR increased with index case age (35–59 years: adjusted OR [aOR], 3.4; 95% CI, 1.5–7.8 and ≥60 years: aOR, 3.1; 95% CI, 1.0–10.1) compared with 18–34 years and with contacts’ age, 13–17 years (aOR, 7.1; 95% CI, 1.5–33.9) and 18–34 years (aOR, 4.4; 95% CI, 1.0–18.4) compared with <5 years. Mean positivity was longer in cases LWH (adjusted hazard ratio, 0.4; 95% CI, .1–.9).

**Conclusions:**

Index HIV status was not associated with higher HCIR, but cases LWH had longer positivity duration. Adults aged >35 years were more likely to transmit and individuals aged 13–34 to be infected SARS-CoV-2 in the household. As HIV infection may increase transmission, health services must maintain HIV testing and antiretroviral therapy initiation.

## Teaser

In this case-ascertained, prospective, household transmission study, household cumulative infection risk was 59% from symptomatic severe acute respiratory syndrome coronavirus 2 (SARS-CoV-2) index cases, not differing based on index human immunodeficiency virus (HIV) status. Index cases living with HIV were positive for SARS-CoV-2 for longer at higher viral loads.

By January 2022, South Africa had reported 3.6 million coronavirus disease 2019 (COVID-19) cases and 94.3 thousand deaths; the highest reported from Africa [1, 2]. South Africa experienced 4 severe acute respiratory syndrome coronavirus 2 (SARS-CoV-2) waves; the first dominated by ancestral variants, followed by Beta (B.1.351), Delta (B.1.617.2), and Omicron (B.1.1.529) variant waves [3].

Although incident human immunodeficiency virus (HIV) infections and acquired immune deficiency syndrome (AIDS)-related deaths from 2010 to 2019 in South Africa declined by 53% and 61%, respectively, the burden of HIV is still high, with an estimated 19% of the adult population aged 15–49 years living with HIV (LWH); the fourth highest in sub-Saharan Africa [4]. The SARS-CoV-2 pandemic impacted several health programs, including HIV testing and care. During initial lockdowns, there was a decline in HIV testing and antiretroviral therapy initiations, which gradually returned to pre-lockdown levels in South Africa [5] and other sub-Saharan African countries [6].

Few studies have reported on the influence of HIV infection on SARS-CoV-2, with most data available from high-income countries and little evidence from sub-Saharan Africa where most people LWH reside [4]. People LWH are at greater risk for hospitalization [7–10] and death [9–13] when infected with SARS-CoV-2, but SARS-CoV-2 prevalence is similar between people LWH and people not living with HIV (NLWH) [14]. Risk for hospitalization and death increases with a decline in CD4+ T cells [8, 9, 13]. Limited data are available on the role of HIV in the transmission of SARS-CoV-2. One study showed no increase in household transmission from or acquisition of SARS-CoV-2 infection in people LWH [2]. People LWH with severe COVID-19 who are not virally suppressed shed SARS-CoV-2 for longer periods [2, 15], which could lead to increased secondary transmission.

We assessed household cumulative infection risk (HCIR), duration of SARS-CoV-2 positivity (episode duration), and serial interval in households with SARS-CoV-2 index cases LWH and NLWH from October 2020 to September 2021 during the Beta and Delta waves.

## METHODS

We conducted a case-ascertained, prospective, observational, household transmission study of household contacts of symptomatic adult index SARS-CoV-2 cases LWH and NLWH at 2 sites in South Africa: Klerksdorp (North West Province) and Soweto (Gauteng Province). Planned sample size was 264 and 176 contacts from households with an index case NLWH and LWH, respectively (see the Supplementary Materials). Actual sample size was 344 and 103 household members exposed to an index case NLWH and LWH, respectively.

### Screening for Index Cases

Screening procedures are detailed in the Supplementary Methods. In short, nasopharyngeal swabs were collected from clinic attendees aged ≥18 years with symptom onset ≤5 days prior to screening and tested for SARS-CoV-2 using real-time reverse-transcription polymerase chain reaction (rRT-PCR).

### Household Enrollment

We approached households of individuals who tested positive for SARS-CoV-2 with symptom onset <7 days prior and no household members reporting symptoms in the 14 days prior to index screening. We enrolled households with ≥3 eligible members (sharing ≥2 meals in the same residence for ≥2 days/week) and where ≥70% of housheold members consented to participate. Households that withdrew within 10 days from index symptom start date were excluded from the analysis.

### Index and Household Follow-up

We visited households 3 times a week for 6 weeks to collect nasal swabs and data on symptoms and healthcare-seeking behavior from consenting household members. At the first and last study visits, clotted blood was collected for serological testing. Follow-up started on 12 October 2020 and continued to 11 August 2021 and 28 September 2021 in Klerksdorp and Soweto, respectively.

### SARS-CoV-2 Detection

Nasopharyngeal (screening) and nasal (follow-up) specimens were tested for SARS-CoV-2 genes using qualitative rRT-PCR with the Allplex 2019-nCoV kit (Seegene Inc, Seoul, South Korea). Specimens were considered positive for SARS-CoV-2 if the cycle threshold (Ct) value was <40 for any gene target.

### SARS-CoV-2 Variants

We characterized the first SARS-CoV-2–positive specimen for each participant using the Allplex SARS-CoV-2 Variants I and II PCR assays (Seegene Inc, Seoul, Korea) and through full genome sequencing on the Ion Torrent Genexus platform (Thermo Fisher Scientific). We classified the infection episodes as Alpha, Beta, Delta, non-Alpha/Beta/Delta, or unknown variant (see the Supplementary Material).

### Serology

We used an in-house enzyme-linked immunosorbent assay to detect antibodies against the SARS-CoV-2 spike protein [16] and nucleocapsid protein using the Roche Elecsys anti-SARS-CoV-2 assay. Individuals were considered seropositive if they tested positive on either.

### Statistical Analyses

Definitions of terms used for this study are listed in Table 1. To assess factors associated with HCIR, we used logistic regression accounting for within-site and household clustering using a mixed-effects hierarchical regression model. To assess factors associated with a time-to-event analysis (serial interval and episode duration), we used a multilevel mixed-effects survival model with Weibull accelerated failure time analysis. Hazard ratios (HRs) <1 correspond to longer episode duration than observed in the reference group. Since multiple members from the same household could potentially be included in the serial interval analysis, we controlled for both site- and household-level clustering in the analysis. In the episode duration analysis, we controlled for only site clustering (1 index per household). In addition to using site to control for clustering, it was also included as a covariate in models. Episode duration was assessed at any Ct value (<40) and Ct <30 (proxy for high viral load based on virus culture studies [17]). We first assessed covariates on univariate analysis, including all with *P* < .2 in the multivariable analysis. We performed backward elimination and kept all variables with *P* < .05 in the final model, except those included a priori. We included site, SARS-CoV-2 variant, and index immune suppression related to HIV status (defined as CD4+ T-cell count <200 cells/mL) in the HCIR and episode duration models a priori irrespective of statistical significance in the multivariable model. Site was included a priori in the serial interval analysis. Due to low SARS-CoV-2 vaccination coverage in study participants (only 1 contact, Table 2), vaccination status was not included in our analyses.

**Table 1.** Definitions of Terms Used for the Study

Household	A group of 3 or more people who regularly share at least 2 meals in the same residence at least 2 days per week (residential institutions excluded).
Index case	The first household member who had coronavirus disease 2019–like symptoms. We assumed that the household member screened was the index case within the household as they were the first household member to develop symptoms.
SARS-CoV-2 infection episode	At least 1 nasal swab rRT-PCR–positive for SARS-CoV-2. Individuals who seroconverted during follow-up but with no rRT-PCR–confirmed infection were not included in secondary case analyses.
SARS-CoV-2 cluster	Composed of all infections within a household within an interval between infections of ≤2 weeks including single infections within a household.
Episode duration	Duration of SARS-CoV-2 positivity. The start of symptom onset to the midpoint between the last positive swab and first negative swab. Individuals who were still SARS-CoV-2 positive on the last study visit (whether at the end of follow-up or due to early withdrawal) were right-censored for the multivariable analysis.
Serial interval	Number of days between the onset of symptoms in the index case and the onset of symptoms in the secondary case. Multivariable analyses were restricted to symptomatic secondary cases and to serial interval periods of ≤21 days as longer serial intervals could have been due to tertiary cases or secondary infections.
HCIR	The percentage of susceptible household members (based on baseline serology) who had at least 1 SARS-CoV-2–positive swab from the start of follow-up up to 2 weeks from the last SARS-CoV-2–positive swab of the index case. Considering susceptibility was important because following the second wave of SARS-CoV-2 infection in South Africa, 41% of individuals were estimated to have had previous SARS-CoV-2 infection [18]. Individuals with SARS-CoV-2 antibodies detected at baseline, but also tested positive on rRT-PCR, were included in the HCIR calculation. Individuals for whom no baseline serology was available were included in the analysis as presumed susceptible. We did not consider any secondary introductions in the household for our analysis. Households where members had SARS-CoV-2 infection with different variants of concern were excluded from the analysis.

Abbreviations: HCIR, household cumulative infection risk; rRT-PCR, real-time reverse-transcription polymerase chain reaction; SARS-CoV-2, severe acute respiratory syndrome coronavirus 2.

**Table 2.** Baseline Characteristics of Severe Acute Respiratory Syndrome Coronavirus 2 Index Cases (n = 131) and Their Household Contacts (n = 457), Klerksdorp and Soweto, South Africa, September 2020–October 2021

	Overall		Index Case, n/N (%)		Household Contact, n/N (%)	
Characteristic	Index Case, n/N (%)	Household Contact, n/N (%)	Klerksdorp	Soweto	Klerksdorp	Soweto
Household characteristics						
Index case/contact	131/588 (22)	457/588 (78)	62/274 (23)	69/314 (22)	212/274 (77)	245/314 (78)
Household size						
ȃ3–5	106/131 (81)	305/457 (67)	51/62 (82)	55/69 (80)	146/212 (69)	159/245 (65)
ȃ6–10	25/131 (19)	152/457 (33)	11/62 (18)	14/69 (20)	66/212 (31)	86/245 (35)
Rooms used for sleeping						
ȃ1–2	66/117 (56)	209/401 (52)	33/57 (58)	33/60 (55)	114/192 (59)	95/209 (45)
ȃ3–4	42/117 (36)	141/401 (35)	22/57 (39)	20/60 (33)	69/192 (36)	72/209 (34)
ȃ>4	9/117 (8)	51/401 (13)	2/57 (4)	7/60 (12)	9/192 (5)	42/209 (20)
Crowding	89/131 (68)	330/457 (72)	43/62 (69)	46/69 (67)	158/212 (75)	172/245 (70)
Child aged <5 years	17/131 (13)	63/457 (14)	10/62 (16)	7/69 (10)	43/212 (20)	20/245 (8)
Household member smokes inside	30/131 (23)	93/457 (20)	18/62 (29)	12/69 (17)	51/212 (24)	42/245 (17)
Main water source inside home	92/131 (70)	321/457 (70)	38/62 (61)	54/69 (78)	124/212 (58)	197/245 (80)
Place to wash hands inside	129/131 (98)	451/457 (99)	62/62 (100)	67/69 (97)	212/212 (100)	239/245 (98)
Main cooking fuel						
ȃElectricity	127/131 (97)	444/457 (97)	58/62 (94)	69/69 (100)	199/212 (94)	245/245 (100)
ȃGas/Paraffin	4/131 (3)	13/457 (3)	4/62 (6)	0/69 (0)	13/212 (6)	0/245 (0)
Monthly household income						
ȃ<US$23	5/131 (4)	17/457 (4)	4/62 (6)	1/69 (1)	15/212 (7)	2/245 (1)
ȃUS$24 to US$46	6/131 (5)	19/457 (4)	4/62 (6)	2/69 (3)	12/212 (6)	7/245 (3)
ȃUS$47 to US$93	12/131 (9)	39/457 (9)	10/62 (16)	2/69 (3)	33/212 (16)	6/245 (2)
ȃUS$94 to US$187	23/131 (18)	86/457 (19)	12/62 (19)	11/69 (16)	43/212 (20)	43/245 (18)
ȃ$US188 to US$375	21/131 (16)	68/457 (15)	7/62 (11)	14/69 (20)	23/212 (11)	45/245 (18)
ȃUS$376 to US$752	8/131 (6)	33/457 (7)	2/62 (3)	6/69 (9)	5/212 (2)	28/245 (11)
ȃUS$753 to US$1506	6/131 (5)	19/457 (4)	0/62 (0)	6/69 (9)	0/212 (0)	19/245 (8)
ȃRefused to disclose	50/131 (38)	176/457 (39)	23/62 (37)	27/69 (39)	81/212 (38)	95/245 (39)
Individual characteristics						
Age, years						
ȃ<5	…	19/457 (4)	…	…	11/212 (5)	8/245 (3)
ȃ5–12	…	80/457 (18)	…	…	39/212 (18)	41/245 (17)
ȃ13–17	…	70/457 (15)	…	…	38/212 (18)	32/245 (13)
ȃ18–34	37/131 (28)	126/457 (28)	21/62 (34)	16/69 (23)	54/212 (25)	72/245 (29)
ȃ35–59	76/131 (58)	113/457 (25)	37/62 (60)	39/69 (57)	50/212 (24)	63/245 (26)
ȃ≥60	18/131 (14)	49/457 (11)	4/62 (6)	14/69 (20)	20/212 (9)	29/245 (12)
Sex						
ȃMale	38/131 (29)	192/457 (42)	17/62 (27)	21/69 (30)	93/212 (44)	99/245 (40)
ȃFemale	93/131 (71)	265/457 (58)	45/62 (73)	48/69 (70)	119/212 (56)	146/245 (60)
Level of education^a^						
ȃNo schooling	6/129 (5)	15/283 (5)	3/60 (5)	3/69 (4)	1/120 (1)	14/163 (9)
ȃPrimary	4/129 (3)	21/283 (7)	2/60 (3)	2/69 (3)	14/120 (12)	7/163 (4)
ȃSecondary	43/129 (33)	89/283 (31)	25/60 (42)	18/69 (26)	47/120 (39)	42/163 (26)
ȃMatriculation	69/129 (53)	136/283 (48)	28/60 (47)	41/69 (59)	51/120 (43)	85/163 (52)
ȃPost-secondary	7/129 (5)	22/283 (8)	2/60 (3)	5/69 (7)	7/120 (6)	15/163 (9)
Employment^a^						
ȃUnemployed	55/121 (45)	159/258 (62)	25/57 (44)	30/64 (47)	68/111 (61)	91/147 (62)
ȃStudent	10/121 (8)	30/258 (12)	5/57 (9)	5/64 (8)	11/111 (10)	19/147 (13)
ȃEmployed	56/121 (46)	69/258 (27)	27/57 (47)	29/64 (45)	32/111 (29)	37/147 (25)
Smoking cigarettes^b^	17/129 (13)	66/323 (20)	11/60 (18)	6/69 (9)	35/145 (24)	31/178 (17)
Smoke indoors	5/17 (29)	15/66 (23)	5/11 (45)	0/6 (0)	14/35 (40)	1/31 (3)
HIV status						
ȃNot living with HIV	101/131 (77)	176/457 (39)	51/62 (82)	50/69 (72)	130/212 (61)	46/245 (19)
ȃLiving with HIV	28/131 (21)	35/457 (8)	10/62 (16)	18/69 (26)	16/212 (8)	19/245 (8)
ȃUnknown	2/131 (2)	246/457 (54)	1/62 (2)	1/69 (1)	66/212 (31)	180/245 (73)
Index case living with HIV in household						
ȃIndex HIV-negative	101/131 (77)	344/457 (75)	51/62 (82)	50/69 (72)	175/212 (83)	169/245 (69)
ȃIndex HIV- positive	28/131 (21)	103/457 (23)	10/62 (16)	18/69 (26)	34/212 (16)	69/245 (28)
ȃIndex HIV unknown	2/131 (2)	10/457 (2)	1/62 (2)	1/69 (1)	3/212 (1)	7/245 (3)
CD4+ T-cell count						
ȃNot living with HIV, not immune suppressed^c^	17/28 (61)	13/35 (37)	5/10 (50)	12/18 (67)	2/16 (13)	11/19 (58)
ȃLiving with HIV, immune suppressed^c^	4/28 (14)	2/35 (6)	1/10 (10)	3/18 (17)	1/16 (6)	1/19 (5)
ȃLiving with HIV, no CD4+ T-cell count	7/28 (25)	20/35 (57)	4/10 (40)	3/18 (17)	13/16 (81)	7/19 (37)
Underlying illness^d^	32/129 (25)	57/448 (13)	12/60 (20)	20/69 (29)	205/212 (97)	243/245 (99)
Body mass index						
ȃUnderweight	3/129 (2)	29/448 (6)	1/60 (2)	2/69 (3)	14/205 (7)	15/243 (6)
ȃNormal	36/129 (28)	206/448 (46)	18/60 (30)	18/69 (26)	108/205 (53)	98/243 (40)
ȃOverweight	25/129 (19)	103/448 (23)	10/60 (17)	15/69 (22)	39/205 (19)	64/243 (26)
ȃObese	65/129 (50)	110/448 (25)	31/60 (52)	34/69 (49)	44/205 (21)	66/243 (27)
Previous TB	7/129 (5)	9/448 (2)	2/60 (3)	5/69 (7)	6/205 (3)	3/243 (1)
Current TB	4/129 (3)	2/448 (0)	2/60 (3)	2/69 (3)	0/205 (0)	2/243 (1)
Coronavirus 2019 vaccination^e^	0/131 (0)	1/457 (0)	0/62 (0)	0/69 (0)	1/212 (0)	0/245 (0)

Abbreviations: HIV, human immunodeficiency virus; TB, tuberculosis.

Individuals aged ≥18 years.

Individuals aged ≥15 years.

Immune suppressed defined as CD4+ T-cell count <200 cells/mL.

Underlying medical conditions include self-reported history of diabetes, hypertension, asthma, lung disease, heart disease, stroke, spinal cord injury, epilepsy, cancer, liver disease, renal disease, prematurity.

At least 1 dose administered 14 days prior to enrollment.

### Sensitivity Analysis

To assess the influence of loss to follow-up, we performed a sensitivity analysis that included only households where 65% of enrolled household members completed 65% of follow-up visits in the first 3 weeks of follow-up. To explore the effect of previous SARS-CoV-2 infection, we considered all household members irrespective of baseline serology as susceptible contacts in the HCIR analysis.

### Ethics

The University of the Witwatersrand Human Research Ethics Committee approved the study protocol. Participants in follow-up received a $3.00 grocery store voucher per visit to compensate for time required for specimen collection and interview.

## RESULTS

### Screening, Enrollment, and Follow-up

**Figure 1.**
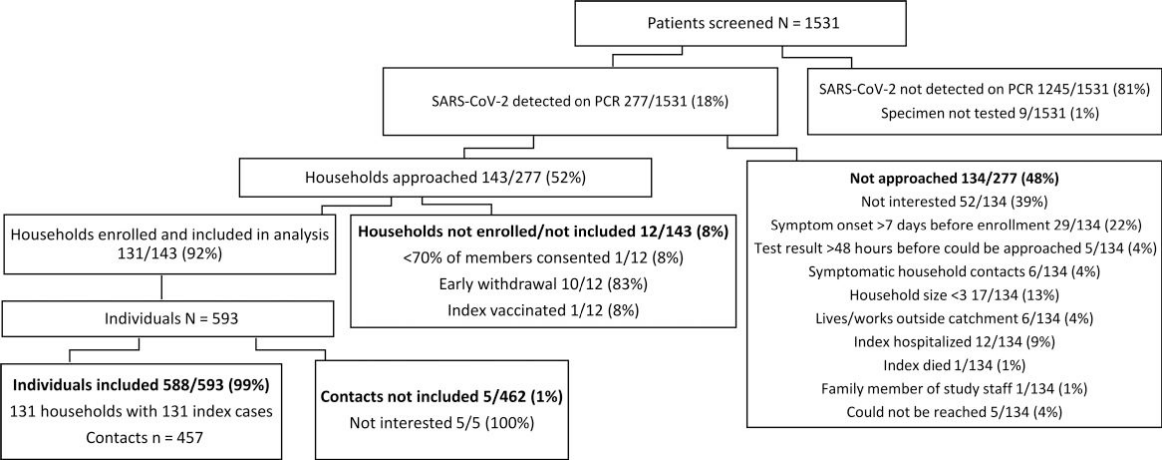
Adult SARS-CoV-2 index cases and household contacts enrolled, Klerksdorp and Soweto, South Africa, 2020–2021. Abbreviations: PCR, polymerase chain reaction; SARS-CoV-2, severe acute respiratory syndrome coronavirus 2.

From 2 October 2020 to 30 September 2021, we screened 1531 clinic attendees for SARS-CoV-2; 18% (277) tested positive on rRT-PCR. Of those who tested positive and met eligibility criteria for household enrollment (n = 277), 143 (52%) households were approached and 131 (92%) were enrolled. Reasons for noninclusion are shown in Figure 1. The final cohort consisted of 131 index cases and 457 household contacts (Figure 1); the median household size was 4.

Twenty-one percent (28 of 131) of index cases were LWH, and 2 index cases initially agreed but then refused HIV testing after enrollment (classified as HIV unknown during analyses). The majority (93 of 131, 71%) of index cases and contacts (265 of 457, 58%) were female (Table 2).

Of 10 584 potential study visits to individual participants, we completed 8509 (80%) visits and detected SARS-CoV-2 in 17% (1454 of 8352) of nasal swabs collected (Supplementary Figure 1).

### Secondary SARS-CoV-2 Cases

We diagnosed 232 (51%) rRT-PCR–confirmed secondary cases from 457 contacts linked to 131 index cases. One-third (69 of 232) of secondary cases reported ≥1 symptom during their SARS-CoV-2 episode, reporting on average 3 symptoms (range, 1–9). The mean symptom duration was 11 days (range, 4–40). The most common symptoms reported were cough (45 of 69, 65%), headache (31 of 67, 46%), and fever (27 of 69, 39%). Five secondary cases were hospitalized (Supplementary Table 5).

### Household Cumulative Infection Risk

Of 131 households, we excluded 7 (5%) from the HCIR analysis: 4 (13 contacts) had SARS-CoV-2 clusters with >1 variant detected (Supplementary Figure 2); in 3 (8 contacts), all contacts were seropositive at baseline with no rRT-PCR–confirmed SARS-CoV-2 infection during follow-up. An additional 42 contacts were excluded because they had prevalent SARS-CoV-2 antibodies at baseline and no SARS-CoV-2 detection during follow-up. We therefore included 124 of 131 (95%) index cases with 373 of 436 (86%) contacts for this analysis.

The HCIR was 59% (220 of 373) overall. The mean number of household contacts who tested positive for SARS-CoV-2 following the index episode was 2 (range, 0 to 7). On univariate analysis, HCIR was similar in households with index cases NLWH (58%, 173 of 293) and households where the index was LWH (60%; 50 of 83; odds ratio [OR], 1.0; 95% confidence interval [CI], .4–2.3).

**Figure 2.**
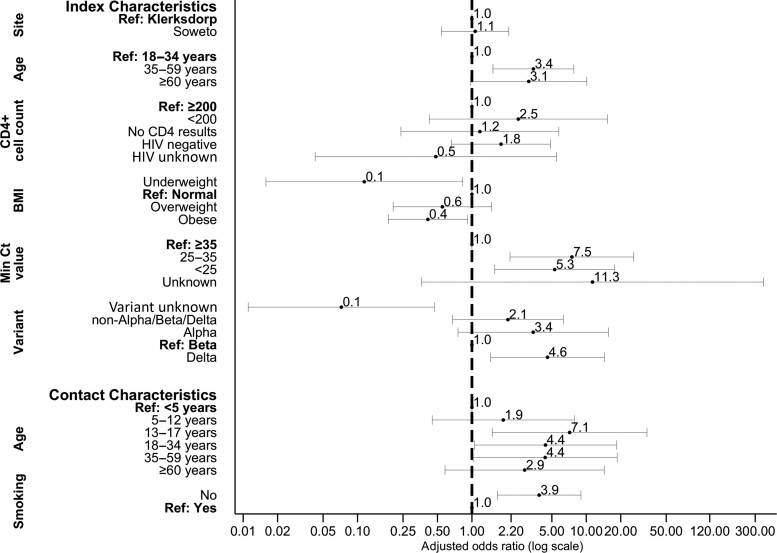
Factors associated with severe acute respiratory syndrome coronavirus 2 household transmission in index cases and acquisition in household contacts on multivariable logistic regression, Klerksdorp and Soweto, South Africa, 2020–2021 (n = 373). Abbreviation: HIV, human immunodeficiency virus.

On multivariable analysis after adjusting for site and immune suppression, factors associated with household transmission were index case aged 35–59 years (adjusted OR [aOR], 3.4; 95% CI, 1.5–7.8) and ≥60 years (aOR, 3.1; 95% CI, 1.0–10.1) compared with 18–34 years; index cases with a Ct value <25 (aOR, 5.3; 95% CI, 1.6–17.6) and 25–35 (aOR, 7.5; 95% CI, 2.2–26.0) compared with Ct >35; and infection with the Delta variant compared with Beta (aOR, 4.6; 95% CI, 1.5–14.4; Figure 2, Supplementary Table 1).

Fourteen percent (17 of 124) of index cases were LWH and not immune suppressed, while 3% (4 of 124) were LWH and immune suppressed. Contacts of index cases LWH with immune suppression had higher HCIR (62%, 8 of 13) compared with contacts of index cases who were not immune suppressed (58%, 30 of 52), but this was not statistically significant on multivariable analysis (aOR, 2.5; 95% CI, .4–15.3). Contact age 13–17 years (aOR, 7.1; 95% CI, 1.5–33.9) and 18–34 years (aOR, 4.4; 95% CI, 1.0–18.4) compared with <5 years and contacts not currently smoking (aOR, 3.9; 95% CI, 1.7–9.0) were associated with higher HCIR.

### Episode Duration

**Figure 3.**
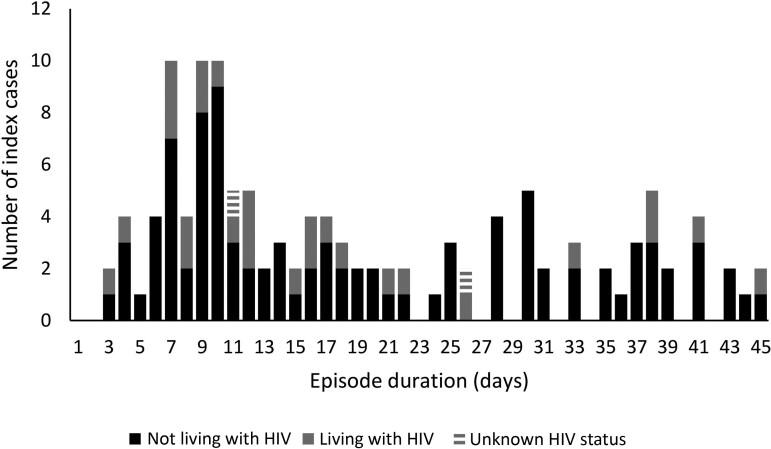
Severe acute respiratory syndrome coronavirus 2 episode duration in index cases living with and not living with HIV, Klerksdorp and Soweto, South Africa, 2020–2021 (n = 123). Excludes those positive at last specimen collected. Abbreviation: HIV, human immunodeficiency virus.

We right-censored 6% (8 of 131) of index cases who were SARS-CoV-2–positive on their last specimen collected at the end of follow-up (n = 5) or at withdrawal from the study (n = 3). When all 131 index cases were included, the mean episode duration for index cases was 20 days (range, 3–47; Figure 3). When we excluded the 8 right-censored individuals (n = 123), the mean episode duration for index cases was 19 days (range, 3 to 45). The mean episode duration was similar for index cases NLWH (20 days; range, 3–45) compared with index cases LWH (17 days; range, 3–45; hazard ratio [HR], 0.8; 95% CI, .5–1.2).

**Figure 4.**
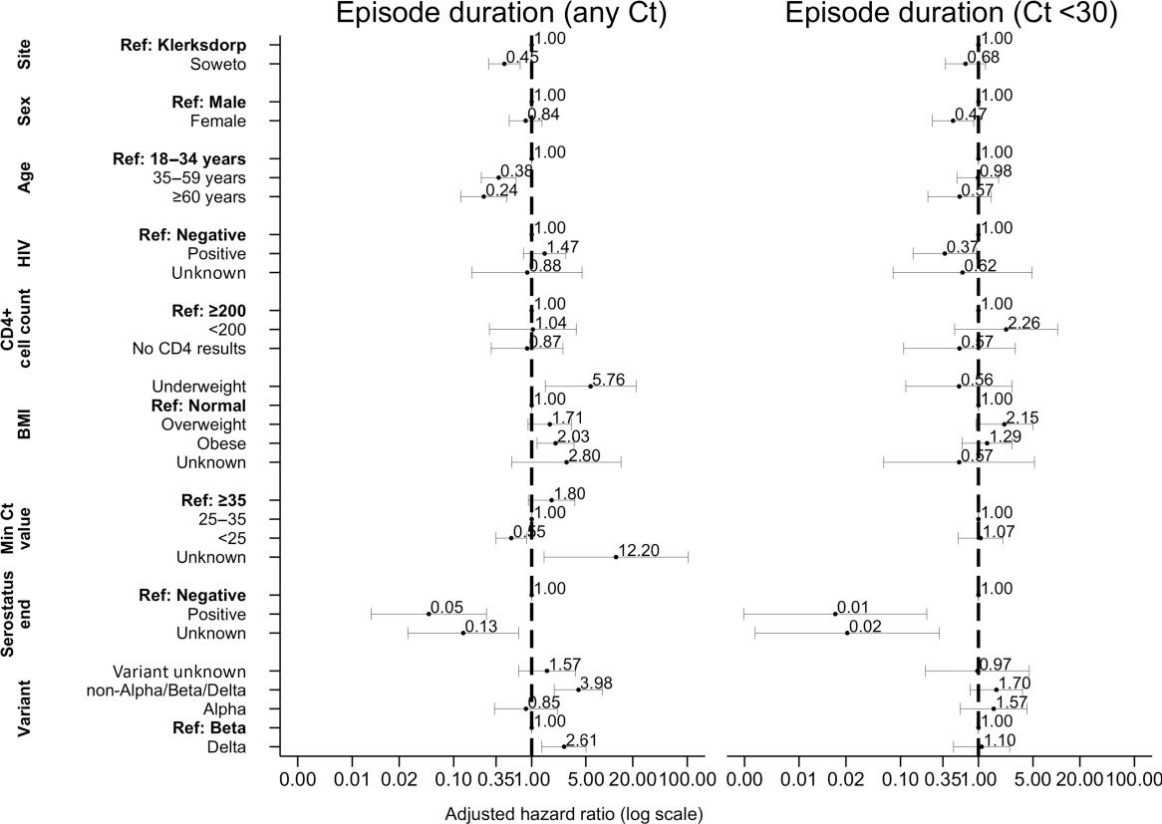
Factors associated with severe acute respiratory syndrome coronavirus 2 episode duration irrespective of Ct value (left), Ct <30 (right) in index cases on Weibull accelerated failure time regression, Klerksdorp and Soweto, South Africa, 2020–2021 (n = 123). Abbreviations: BMI, body mass index; Ct, cycle threshold; HIV, human immunodeficiency virus.

On multivariable analysis, factors associated with longer episode duration at any Ct value, in days, were Soweto site (adjusted HR [aHR], 0.5; 95% CI, .3–.7); being aged 35–59 years (aHR, 0.4; 95% CI, .2–.6) and being aged ≥60 years (aHR, 0.2; 95% CI, .1–.5) compared with aged 18–34 years; Ct <25 (aHR, 0.6; 95% CI, .2–.9) compared with Ct >35 (Figure 4, Supplementary Table 2); and being seropositive at the end of follow-up (aHR, 0.1; 95% CI, .0–.3). Individuals infected with the Delta variant (aHR, 2.6; 95% CI, 1.4–5.0) and a nonvariant of concern (aHR, 4.0; 95% CI, 1.9–8.2) compared with Beta had shorter episode durations (Figure 4, Supplementary Table 2).

Eighty-eight (67%) index cases had ≥1 specimen with ≥1 target with Ct <30. Mean episode duration with Ct <30 was 7 days (range, 2–17). On multivariable analysis, factors associated with longer episode duration considering only specimens with at least 1 target with a Ct <30, was female sex (aHR, 0.5; 95% CI, .3–.9), LWH (aHR, 0.4; 95% CI, .1–.9), and being seropositive at the end of follow-up (aHR, 0.01; 95% CI, .001–.2; Figure 4, Supplementary Table 3).

### Serial Interval

**Figure 5.**
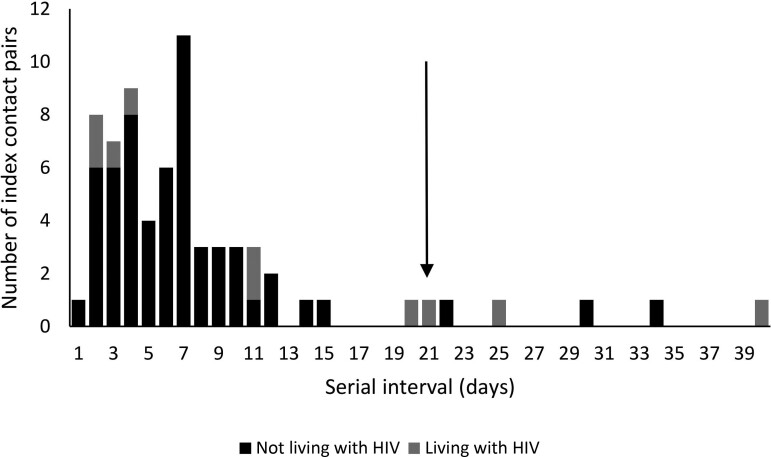
Interval between onset of symptoms in the index case and onset of symptoms in household contacts with severe acute respiratory syndrome coronavirus 2 (serial interval) by HIV status of the index case, Klerksdorp and Soweto, South Africa, 2020–2021 (n = 69). Arrow indicates cutoff for inclusion in analysis for factors associated with serial interval duration. Abbreviations: BMI, body mass index; Ct, cycle threshold; HIV, human immunodeficiency virus.

We excluded 5 index contact pairs where serial interval was >21 days; 3 with an index NLWH and 2 pairs where the index was LWH. Mean serial interval for index cases and symptomatic contact pairs included in the risk factor analysis was 6 days (range, 1–20; Figure 5). Mean serial interval for index cases NLWH was 6 days (range, 1–15); for index cases LWH it was 8 days (range, 2–20).

**Figure 6.**
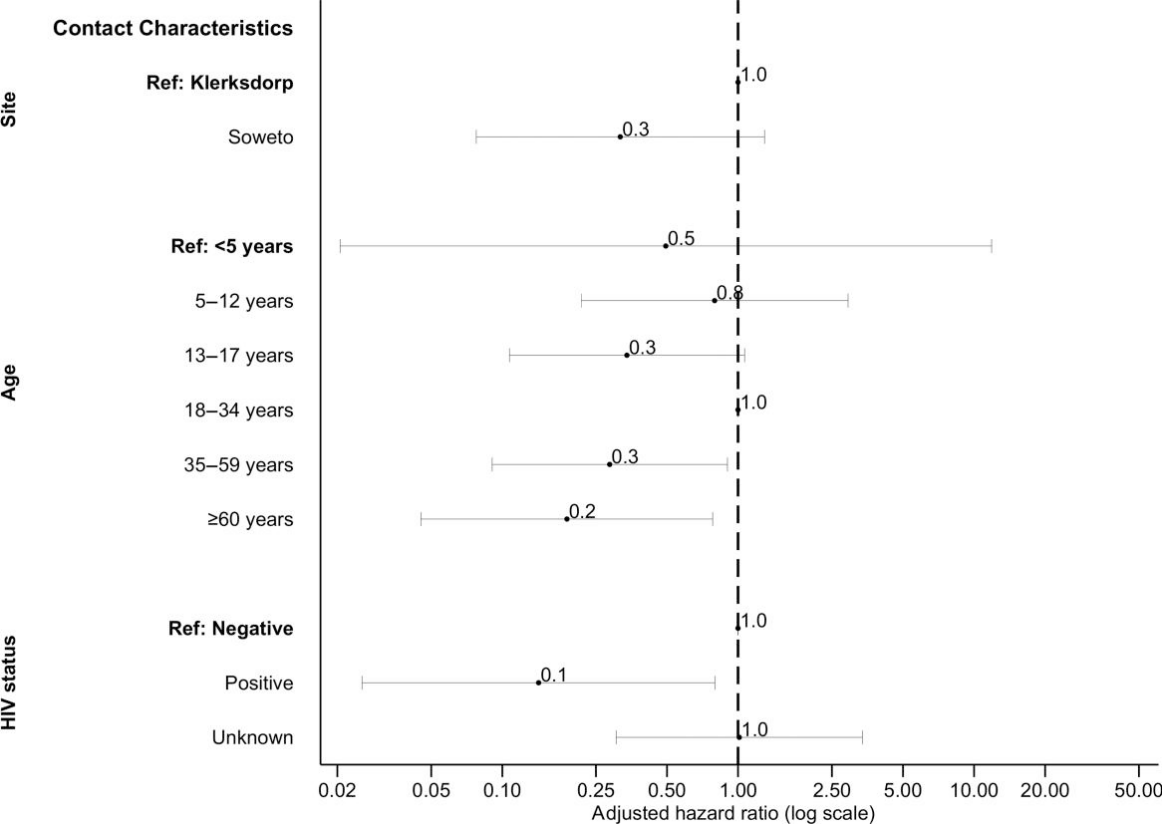
Factors associated with serial interval of severe acute respiratory syndrome coronavirus 2 in cases with symptomatic illness on Weibull accelerated failure time regression, Klerksdorp and Soweto, South Africa, 2020–2021 (n = 62). Abbreviation: HIV, human immunodeficiency virus.

On multivariable analysis, pairs with contacts aged 35–59 years (aHR, 0.3; 95% CI, .1–.9) and ≥60 years (aHR, 0.2; 95% CI, .0–.8) compared with aged 18–34 years and where the contact was LWH (aHR, 0.1; 95% CI, .0–.8) had longer serial intervals (Figure 6, Supplementary Table 4).

### Sensitivity Analysis

When individuals seropositive at baseline with no rRT-PCR–confirmed SARS-CoV-2 infection during follow-up were not excluded, HCIR was 51% (220 of 436) overall. We found similar factors associated with HCIR (Supplementary Table 6). When only households where 65% of members completed 65% of visits were included, we included 112 index cases with 342 contacts. Factors associated with HCIR, episode duration, and serial interval were similar to what we observed in the main analysis (Supplementary Tables 7–9).

## DISCUSSION

We performed a case-ascertained, prospective, household transmission study for SARS-CoV-2 in South Africa, including 131 index cases, 28 of whom were LWH, and 457 household contacts. We observed a 59% HCIR, HCIR being higher in households with older index cases and contacts aged 13–17 years and 18–34 years. HCIR was also higher in households with Delta-infected index cases vs Beta. The HCIR was similar in index cases LWH and NLWH. Index episode durations were longer in older individuals. Episode duration at high viral load (Ct <30) was longer in index cases LWH, and serial interval was longer in contacts LWH.

HCIR from previous studies has varied based on study design, symptom status of the index case, timing within the epidemic [19], and SARS-CoV-2 variant [20]. In our study, which included only symptomatic index cases, we estimated the HCIR at 59%, a higher estimate than the overall 37% reported from a recent meta-analysis that included 33 studies performed in 2021 and 2022 and the variant-specific 23% and 30% estimates for Beta and Delta variants, respectively [20]. In a study from Madagascar that included both symptomatic and nonsymptomatic index cases, HCIR was 39% [21]. The higher estimate seen in our study may be influenced by symptom severity, as proposed in previous studies [22], or the inclusion of only adult index cases; adult index cases result in higher HCIR [23]. In our study, households with index cases aged >35 years were 3 times more likely to result in higher HCIR compared with when index cases were aged 18–34 years. HCIR was also higher when contacts were aged 13–17 and 18–34 years compared with <5 years. Contacts aged 13–18 years were also associated with higher HCIR in the prospective household cohort study of SARS-CoV-2, influenza, and respiratory syncytial virus community burden, transmission dynamics, and viral interaction in South Africa (PHIRST-C) [2], although studies from earlier in the pandemic showed higher attack rates in elderly household members [22, 24]. This may be related to the shift in age distribution of cases from the older population to younger individuals with progression of the pandemic [2, 18, 25]. As seen previously [2, 26, 27], we also observed higher secondary attack rates where the minimum rRT-PCR Ct was lower for the index case, which could be considered a proxy for higher viral load.

We observed no difference in HCIR in households with index cases living with and without HIV. However, we observed a higher HCIR in people LWH who were immune suppressed, but this association was not statistically significant, possibly due to low numbers (n = 14) of included immunosuppressed index cases LWH. This would fit with previous studies that found that immunocompromised people LWH shed virus at low Ct values for longer [2, 15], allowing more opportunity for secondary infections, although we did not observe increased transmission in our study, possibly due to small numbers.

The mean episode duration in index cases of 19 days was higher than the 11 days reported from the household cohort study from South Africa [2] but similar to the 18-day estimate from a meta-analysis for viral shedding time [28]. This may be because our analysis was limited to symptomatic individuals who were shown to be associated with longer episode duration [2, 28]. Episode duration in our study was also longer than in studies of hospitalized South African patients where median episode duration was 13 days [15]. Previous studies from South Africa in the community and in hospitals found that immunocompromised people LWH shed SARS-CoV-2 for longer [2, 15]. While we did not find overall longer shedding in people LWH, when considering detection at Ct <30 (proxy for high viral load), we also observed that people LWH had longer episode durations. Longer episode durations may allow increased opportunity for viral evolution and the establishment of novel variants [29].

Previous serial interval estimates ranged from 4 to 7.5 days [2, 22], similar to our estimate of 6 days. We did not find any index characteristics related to longer serial intervals, but longer serial intervals were observed in contacts aged >35 years and those LWH. Individuals with compromised immune systems (people LWH, the elderly) may still be able to be infected with SARS-CoV-2 toward the end of the index episode when viral loads are lower. Due to their increased risk for hospitalization and death [9, 13], there should be continued support for prioritizing COVID-19 vaccination in these populations.

Our study had limitations. We assumed the first household member who presented with symptoms was the index case. If the true index cases were asymptomatic, we would have underestimated the serial interval, although HCIR estimates should not be greatly affected. Due to the delay between index screening and household enrollment, we did not have the exact date of first SARS-CoV-2 positivity in household contacts and may have also overestimated HCIR if there were multiple introductions of SARS-CoV-2 in the household. By excluding individuals who were seropositive at baseline with no SARS-CoV-2 infection during follow-up, we assumed 100% protection from previous infections, which is likely not correct, and may have overestimated HCIR. When these individuals were included, HCIR reduced by 8%. We were unable to reach the planned sample size for contacts of index cases LWH and may have been underpowered to detect some differences. By only including symptomatic index cases, our results may not be generalizable to households with asymptomatic infections. We did not consider the role of age-related contact patterns within the household on household transmission. This should be considered for future studies.

In conclusion, in 2 communities in South Africa, HCIR was higher than in previous studies [20] and not influenced by HIV status. Episode duration at high viral loads (inferred by Ct <30) was increased for index cases LWH, which may lead to increased risk for secondary transmission and viral evolution. Serial interval was longer in contacts LWH. Although these findings indicate that HIV status of the index case did not affect SARS-CoV-2 transmission to household contacts, it may still play a role, especially if people LWH are not virally suppressed. Sustaining and strengthening HIV treatment and care programs should be a focus moving forward to ensure people LWH are diagnosed and virally suppressed to reduce prolonged shedding of SARS-CoV-2 and potentially reduce increased transmission, as well as their risk for hospitalization and death [9].

## Supplementary Data

Supplementary materials are available at *Clinical Infectious Diseases* online. Consisting of data provided by the authors to benefit the reader, the posted materials are not copyedited and are the sole responsibility of the authors, so questions or comments should be addressed to the corresponding author.

## Supplementary Material

ciac640_Supplementary_Data
